# Transcriptome Profiles of Strawberry (*Fragaria vesca*) Fruit Interacting With *Botrytis cinerea* at Different Ripening Stages

**DOI:** 10.3389/fpls.2019.01131

**Published:** 2019-09-18

**Authors:** Zeraye Mehari Haile, Ellaine Grace Nagpala-De Guzman, Marco Moretto, Paolo Sonego, Kristof Engelen, Lisa Zoli, Claudio Moser, Elena Baraldi

**Affiliations:** ^1^Laboratory of Biotechnology and Plant Pathology, DISTAL, University of Bologna, Bologna, Italy; ^2^Plant Protection Research Division of Melkasa Agricultural Research Center, Ethiopian Institute of Agricultural Research (EIAR), Addis Ababa, Ethiopia; ^3^Genomics and Biology of Fruit Crops Department, Research and Innovation Centre, Fondazione Edmund Mach, San Michele all’Adige, Italy; ^4^Unit of Computational Biology, Research and Innovation Centre, Fondazione Edmund Mach, San Michele all’Adige, Italy; ^5^ESAT-ELECTA, Electrical Energy and Computer Architectures, Leuven, Belgium

**Keywords:** Botrytis cinerea, defense response, Fragaria vesca, fungal quiescence, transcriptomic analysis

## Abstract

Gray mold caused by *Botrytis cinerea* is a major cause of economic losses in strawberry fruit production, limiting fruit shelf life and commercialization. When the fungus infects *Fragaria* × *ananassa* strawberry at flowering or unripe fruit stages, symptoms develop after an extended latent phase on ripe fruits before or after harvesting. To elucidate the growth kinetics of *B. cinerea* on flower/fruit and the molecular responses associated with low susceptibility of unripe fruit stages, woodland strawberry *Fragaria vesca* flowers and fruits, at unripe white and ripe red stages, were inoculated with *B. cinerea*. Quantification of fungal genomic DNA within 72 h postinoculation (hpi) showed limited fungal growth on open flower and white fruit, while on red fruit, the growth was exponential starting from 24 hpi and sporulation was observed within 48 hpi. RNA sequencing applied to white and red fruit at 24 hpi showed that a total of 2,141 genes (12.5% of the total expressed genes) were differentially expressed due to *B. cinerea* infection. A broad transcriptional reprogramming was observed in both unripe and ripe fruits, involving in particular receptor and signaling, secondary metabolites, and defense response pathways. Membrane-localized receptor-like kinases and nucleotide-binding site leucine-rich repeat genes were predominant in the surveillance system of the fruits, most of them being downregulated in white fruits and upregulated in red fruits. In general, unripe fruits exhibited a stronger defense response than red fruits. Genes encoding for pathogenesis-related proteins and flavonoid polyphenols as well as genes involved in cell-wall strengthening were upregulated, while cell-softening genes appeared to be switched off. As a result, *B. cinerea* remained quiescent in white fruits, while it was able to colonize ripe red fruits.

## Introduction

Strawberry ripening is characterized by the simultaneous changes in the physical and chemical structure of the fruit such as the size, color, flavor, aroma, and texture (Schwab and Raab, 2004; Liao et al., 2018). From the inflorescence, the receptacle develops into the edible part of the fruit and increases in size as determined by the volume and expansion of cells (Perkins-Veazie, 1995). The fruit color changes from green to white and ultimately turns red as a function of chlorophyll degradation and increased anthocyanin synthesis. Soluble solids and volatile organic compounds which confer flavor and aroma also increase with ripening (Manning, 1993; Perkins-Veazie, 1995; Montero et al., 1996; Schwab and Raab, 2004). Meanwhile, fruits of *Fragaria* × *ananassa* soften with ripening due to the solubilization and depolymerization of cell wall components in strawberry (Rosli et al., 2004; Payasi et al., 2009). As a nonclimacteric crop, the significance of abscisic acid (ABA) as a key hormone in the ripening of *F.* × *ananassa* has been demonstrated and recognized (Jia et al., 2011; Symons et al., 2012), and recent studies have suggested that ABA is important in the regulation of ripening-related genes in strawberry (*F.* × *ananassa*) *via* perception and signal transduction (Li et al., 2011).

Along with the changes that occur during ripening is the decline of the innate immunity of crops against pathogen infection (Amil-Ruiz et al., 2011). The disassembly of cell walls that act as a structural barrier against invading organisms during ripening contributes to making strawberry (*F.* × *ananassa*) fruits susceptible to pathogen attack (Guidarelli et al., 2011). Meanwhile, secondary plant metabolites which are essential in determining nutritional and sensory characteristics of fruits also functions as preformed defense compounds against several pathogens. Concentrations of specific antifungal compounds have been found to decrease from the unripe to the ripe fruit stages of *F.* × *ananassa* (Puhl and Treutter, 2008; Guidarelli et al., 2011; Nagpala et al., 2016). The volatile compounds that are abundantly present in red fruits of *F.* × *ananassa* were also found to be beneficial for pathogen growth (Neri et al., 2015).

*Botrytis cinerea* is one of the most destructive pre- and postharvest strawberry pathogens causing gray mold rot (Feliziani and Romanazzi, 2016), and it is regarded as the second most important fungal pathogen worldwide (Dean et al., 2012). The fungus is a cosmopolitan pathogen able to infect a wide range of plant species (Elad et al., 2016). Moreover, while interacting with resistant unripe fruit, the pathogen is capable of developing quiescent infections, stopping its growth for extended time, and leading to disease symptoms only upon senescence/ripening of host tissues (van Kan, 2005; Prusky et al., 2013; Feliziani and Romanazzi, 2016).

In strawberry (*F.* × *ananassa*), early infection of *B. cinerea* may occur in flowers but remains suppressed until fruit ripening (Bristow et al., 1986; Bulger et al., 1987). It is suggested that the high concentrations of preformed antifungal compounds in the inflorescence of *F.* × *ananassa* are responsible for the inactivity of *B. cinerea* in floral tissues (Terry et al., 2004). Phenotypic evidence of *B. cinerea* quiescencein *F.* × *ananassa* fruits has been demonstrated by artificial inoculation of unripe white and ripe red fruits. Symptoms of infection became evident on red fruits, while white fruits were symptom-free for at least 2 weeks. The advancement of the disease in white fruits 2 weeks after infection is accompanied by evident fruit softening (Nagpala et al., 2016). Meanwhile, elevated concentration of proanthocyanidins, ellagitanins, and catechins were also observed in developing fruits of *F.* × *ananassa* inoculated with *B. cinerea* (Puhl and Treutter, 2008; Nagpala et al., 2016).

The ripening stage of the fruit during which the pathogen established infection largely determines the quiescence of *B. cinerea.* Unripe strawberry fruits are said to be resistant to infection due to the aforementioned innate immunity that could suppress pathogen growth. On the other hand, ripe strawberry fruits provide favorable conditions for the pathogen to resume activity (Petrasch et al., 2019). While some important physical and biochemical aspects related to the ontogenic resistance of strawberry fruits has been explored, several transcriptional mechanisms remain unknown. In recent years, the application of microarray or RNA-Seq allows for a comprehensive and precise elucidation of the gene expression in a given sample. Transcriptome-wide profiling applied to investigate host–pathogen interactions highlights those transcripts regulated upon pathogen infection. Specific studies focused on the *Arabidopsis* response to *B. cinerea* (AbuQamar et al., 2016; Sham et al., 2017) and on the quiescence of fungal pathogens in different development stages of apple leaves (Gusberti et al., 2013), tomato fruits (Alkan et al., 2015), and grapevine fruitlets (Haile et al., 2017) provided stage-specific transcriptome profiles likely related to ontogenic resistance and pathogen activity. A recent RNA-Seq analysis on ripe strawberry (*F.* × *ananassa*) fruits infected with *B. cinerea* reported the time-expression profile of genes from the pathogen and the host during the interaction. Significant changes in the ripe fruit tissues were noted 24 h after *B. cinerea* inoculation, with emphasis on genes involved in pathogen recognition and signal transduction (Xiong et al., 2018).

In the present study, the transcriptome profiles of the unripe white and ripe red fruits of woodland strawberry (*Fragaria vesca*) inoculated with *B. cinerea* were analyzed by RNA-Seq technology in an attempt to elucidate ontogenic resistance mechanisms of strawberry to pathogen attack. The study also provides insights on the gene expression variation between two ripening stages of a nonclimacteric fruit infected with *B. cinerea*. The findings of this study provide useful information to improve the resistance of strawberry fruits against *B. cinerea*, an essential factor to minimize losses caused by the gray mold rot along the strawberry supply chain.

## Materials and Methods

### Pathogen Inoculum and Plant Material

*B. cinerea* (isolate B05.10) was cultured on potato dextrose agar at 21°C, with a photoperiod of 12 h using near ultraviolet light. Meanwhile, potted plants of *F. vesca* cv. “Alpine” were grown under controlled conditions at 22°C with natural light (on average 14 h light during April/May). Conventional management practices were observed, and the plants were maintained pesticide free.

To inoculate strawberry flowers and fruits, conidial suspension of *B. cinerea* was prepared from a 12-day-old culture. The conidia concentration of the suspension was adjusted to 1 × 10^5^ ml^−1^ using a hemacytometer. Open flowers [7 days after anthesis (DAA)] and white (14 DAA) and red (21 DAA) fruits were used. The flowers were inoculated by dropping 10 μl of the conidial suspension on the base of receptacle, while the fruits were inoculated on the fruit surface, toward the base. The same procedure was followed for the control treatment, where distilled water was used instead. The inoculation was made on flowers and fruits intact to its mother plant. After inoculation, the whole plant was bagged in a water-sprayed, clear plastic bag for 24 h to ensure high humidity.

### Growth Kinetics of *B. Cinerea* on *F. Vesca* Flower and Fruit

To investigate the growth of *B. cinerea* in different tissues of strawberry, flower, white fruit, and red fruit were inoculated. Three biological replicates were used for each tissue, where each replicate was composed of 10 open flowers and 5 white and red fruits. Samples were collected at 0, 24, 48, and 72 h postinoculation (hpi), and samples were immediately frozen in liquid nitrogen and kept at −80°C until use. Flower samples, including petals, calyx, stamens, and pistils, were frozen, while fruits were frozen with achenes, without calyx and pedicel.

DNA of *B. cinerea* was extracted from homogenized strawberry tissue and from 2-week-old mycelium with fungal DNA kit (NucleoSpin Plant II, MN), following the manufacturer’s instructions. On the other hand, DNA extraction from strawberry samples was carried out using a cetyl trimethylammonium bromide (CTAB) method, as described by Porebski et al. (1997), with modifications (**Supplemental Information 1**). DNA from mycelium and uninoculated red strawberry fruits were used to generate calibration curves to estimate the amount of fungal DNA in inoculated samples.

Using the genomic DNA as a template, quantitative polymerase chain reaction (qPCR) assays were carried out with the MX3000 thermocycler (Stratagene, CA, USA) to amplify target regions *B. cinerea* and *F. vesca*. Primers targeting the ribosomal region between 28S and 18S genes (intergenic spacer), *Bc3*, were used for *B. cinerea*, while primers targeting the elongation factor gene, *EF*, were used for *F. vesca* (**Supplemental Table 1**). A total of 12.5 µl reaction volume was prepared containing 2.5 µl DNA with Maxima^®^ SYBR Green/ROX qPCR Master Mix (2X; Fermentas) and 200 nM of specific forward and reverse primers. In place of the DNA template, sterile water was used for the negative control. The cycling parameters were as follows: 5 min at 95°C, 40 cycles of 15 s at 95°C, 25 s at 61°C, and 30 s at 72°C. A melting curve was established from 55 to 90°C by changing 0.5°C every 10 s. The efficiency (E) of the PCR assay was calculated using the formula, E = (10 − 1/slope − 1) × 100, where the slope was extracted from the curve Ct = f(log Q0) and Q0 is the initial DNA in the assay. E was expressed as percentage. A fivefold dilution series of genomic DNA of the pathogen and the fruit were used to carry out qPCR reaction to create standard curve by plotting the log value of the starting concentration of DNA (ng) versus the Ct value (**Supplemental Figure 1**). Eventually, the Ct values of the target DNA from the inoculated samples were used to quantify the initial amount of genomic DNA through extrapolation to its corresponding standard curve. The method was applied for both *B. cinerea* and *F. vesca*. The growth of the fungal pathogen in strawberry tissue was analyzed by normalizing the *Botrytis* DNA concentration to the amount of strawberry genomic DNA in that sample. Fungal growth was expressed as picogram of *Botrytis* DNA/nanogram of strawberry DNA.

### RNA Sequencing, Data Processing, and Data Analysis

Samples of white and red fruits, inoculated or mock-inoculated, in three biological replicates were collected at 24 hpi and subjected to RNA sequencing. Total RNA was extracted from frozen samples with mortar and pestle following the protocol described by Lopez-Gomez and Gomez-Lim (1992).

Next generation sequencing of the RNA samples, including sample quality control, was performed by Genomix4life S.R.L. (Baronissi, Salerno, Italy). Indexed libraries were prepared from 2 μg of each purified RNA with TruSeq RNA Sample Prep Kit (Illumina) according to the manufacturer’s instructions. Libraries were quantified using the Agilent 2100 Bioanalyzer (Agilent Technologies) and pooled such that each index-tagged sample was present in equimolar amounts, with a final concentration of 2 nM for the pooled samples. The pooled samples were subjected to cluster generation and sequencing using an Illumina HiSeq 2500 System (Illumina) in a 2 × 100 paired-end format at a final concentration of 8 pM. All raw RNA‐Seq read data are deposited in the National Center for Biotechnology Information (NCBI) Short Read Archive (http://www.ncbi.nlm.nih.gov/sra/) under the BioProject accession code PRJNA530684.

The raw sequence files generated underwent quality control analysis using FastQC Version 0.10.1 (http://www.bioinformatics.babraham.ac.uk/projects/fastqc/), and the Illumina paired-end reads were preprocessed for both quality and adapter trimming with fqtrim Version 0.94 was used with default settings (-A -125 parameters) in order to avoid any read data loss due to false positive (https://ccb.jhu.edu/software/fqtrim/index.shtml). A default setting with the Subread aligner Version 1.4.6 (Liao et al., 2013) was also used for the preprocessed reads to align with the *F. vesca* genome v1.0 assembly (Shulaev et al., 2011). Raw read counts were extracted from the Subread alignments using the feature Count Version 1.4.6 read summarization program (Liao et al., 2014).

The differential expression analysis was performed with the voom method (Law et al., 2014), which estimates the mean–variance relationship of the log counts, generating a precision weight for each observation that is fed into the limma Version 3.23 empirical Bayes analysis pipeline (Smyth, 2004). Differentially expressed genes (DEGs) of *F. vesca* were filtered using a threshold of absolute fold change of log2 ≥ 0.8 with *p* ≤ 0.05. The thresholds were selected based on the expression distribution of each gene, as depicted in volcano plots (**Supplemental Figure 2**). Gene ontology (GO) and functional annotations were assigned based on *F. vesca* version 1.0 hybrid gene models (https://www.rosaceae.org/species/fragaria/fragaria_vesca/genome_v1.0; Shulaev et al., 2011) and Blast2GO (e-value 10^−3^) (Conesa et al., 2005). Functional enrichment was analyzed with AgriGO analysis tool (false discovery rate ≤0.05; Du et al., 2010), using customized annotation and annotated reference of GO terms. MapMan tool (Thimm et al., 2004) was used to visualize DEGs in the context of biotic stress pathway category.

### Quantitative Real-Time PCR Validation of RNA-Seq Data

The expression level of eight randomly selected genes was analyzed to validate RNA-Seq results (**Supplemental Table 1**). cDNA of *F. vesca* was generated from 1 μg of RNA using ImProm-II Reverse TranscriptaseTM (Promega, USA), following the protocol. Amplification of the cDNA was performed with MX3000 thermocycler (Stratagene, CA, USA), utilizing the same proportions of the mix and cycling parameters described in the growth kinetics trial. Quantification was carried out using the relative standard curve method (Applied Biosystems, 1997 updated 2001). Resulting expression of target genes was normalized with *elongation factor* (*EF*) housekeeping gene.

## Results and Discussion

### *Botrytis Cinerea* Infection of Strawberry Flower and Fruit

**Figure 1:**
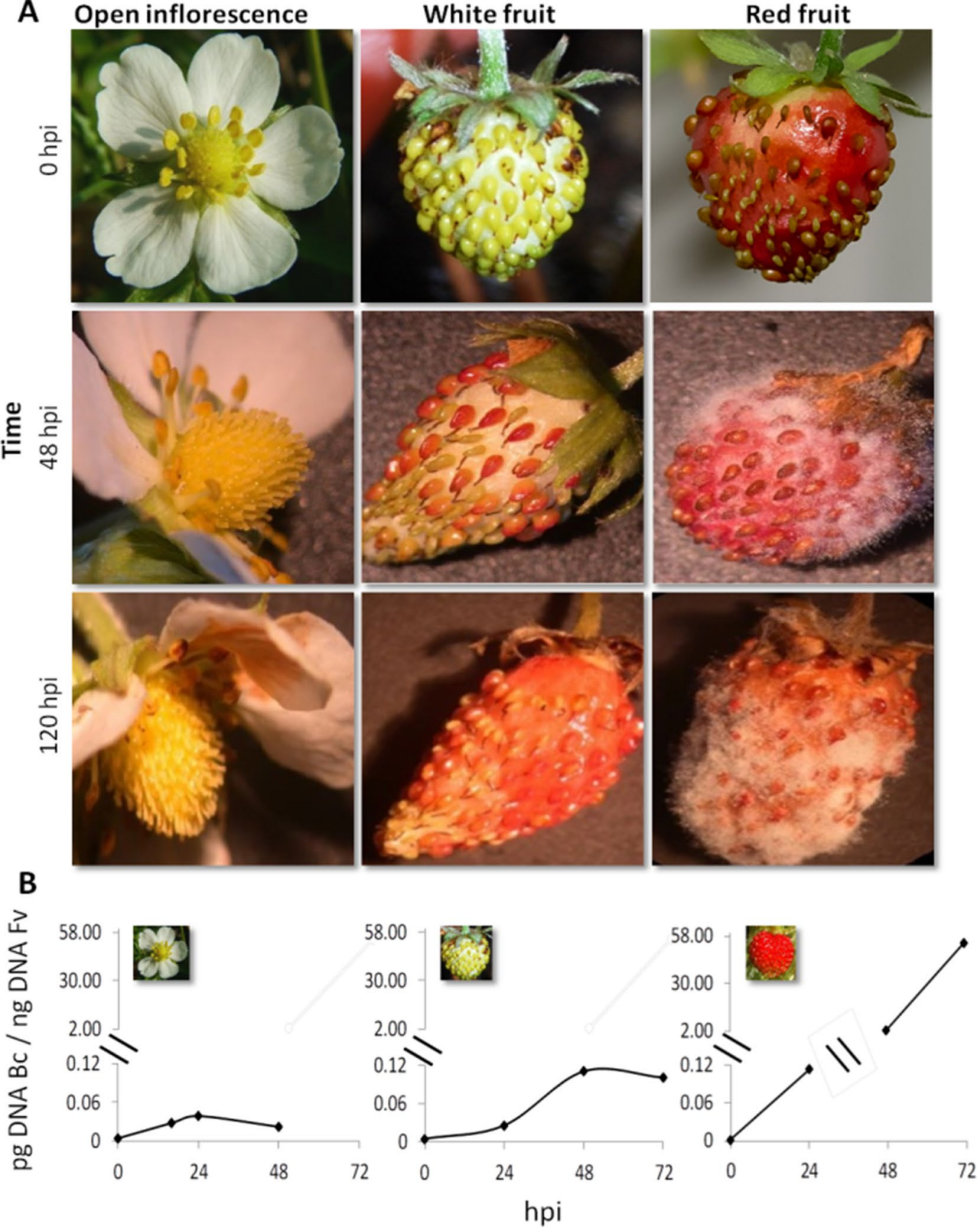
*Botrytis cinerea* growth on *Fragaria vesca* flower and fruits. **(A)** Progress of *B. cinerea* infection on flower, white fruit, and red fruit up to 120 h postinoculation (hpi). Inoculation was made by dropping 10 μl of 1 × 10^5^ ml^−1^ conidial suspension. **(B)** Growth kinetics of *B. cinerea* on flower, white fruit, and red fruit of *F. vesca*. Bc, *B. cinerea*; Fv, *F. vesca*.

Strawberry plants (*F. vesca*) at the stage of open flower, white fruit, and red fruit were inoculated with 1,000 conidia of *B. cinerea* strain B05.10 (**Figure 1**). Infection symptoms were already clearly visible on red fruit at 2 days postinoculation (dpi) and became very severe at 5 dpi (**Figure 1A**). No symptoms of *Botrytis* infection were detected on open flowers(except for petals where brown decay was visible) and white fruits up to 5 dpi (**Figure 1A**). A time-course real-time PCR quantification of *Botrytis* genomic DNA was also performed on the inoculated flowers and fruits to measure the real spread of the fungus also at the early stages after inoculation, in the absence of visible symptoms. Consistent with the phenotype observations, very limited increase in fungal DNA was detected on open flowers and white fruits, and only a very minor growth occurred up to 48 hpi. Conversely, in red fruits, *Botrytis* showed an exponential growth starting at 24 hpi (**Figure 1B**). This indicates that i) receptacles in open flowers or white stages are much less susceptible to *B. cinerea* infection and ii) possibly the pathogen infecting at these stages may become quiescent. Similarly, resistance of flowers and fruitlets of grapevine to *B. cinerea* was also observed where *Botrytis*, inoculated at cap-off stage, remained quiescent for 12 weeks before egression took place at ripening (Haile et al., 2017).

So far, ontogenic variations of polyphenol biosynthesis in strawberry during fruit ontogenesis have been indicated as the basis for quiescence/infection of *B. cinerea* (Jersch et al., 1989; Puhl and Treutter, 2008; Nagpala et al., 2016). This study provides a molecular understanding to the response of unripe and ripe strawberry fruits to *B. cinerea* infection, at the whole transcriptome level.

### RNA-Seq Analysis of *Botrytis*-Inoculated White and Red Strawberry Fruits

**Figure 2:**
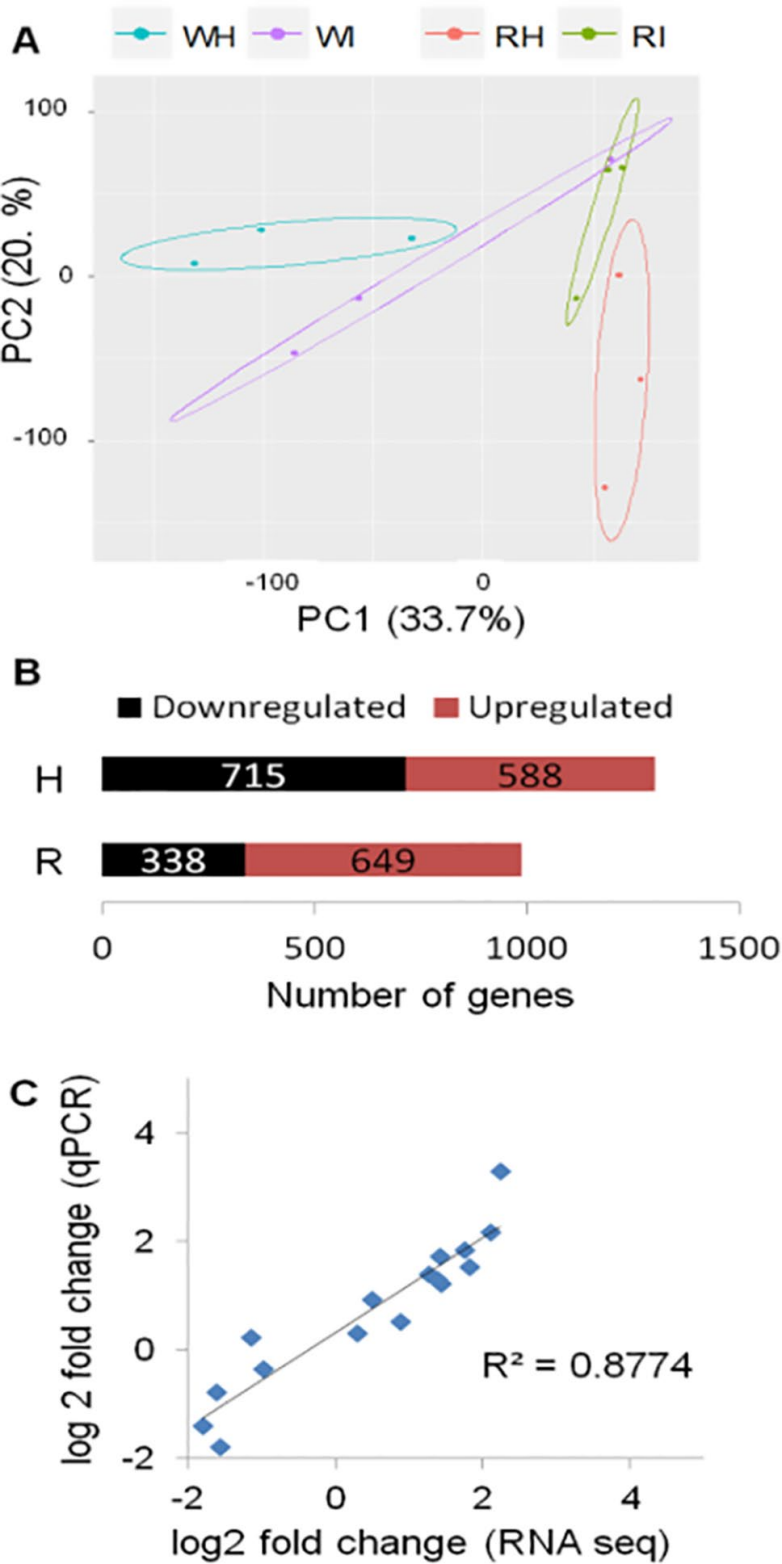
Global evaluation of the RNA-seq data and of the differentially expressed (DE) genes at 24 h postinoculation (hpi). **(A)** Principal component analysis displaying the biological variations of *Fragaria vesca* genes among samples. W, white fruit; R, red fruit; H, healthy (mock inoculated); I, *Botrytis cinerea* inoculated. Raw count data were used after precision weight was calculated by the voom method (Law et al., 2014). **(B)** Number of DE genes (*P* < 0.05, absolute fold change >1.74) upon *B. cinerea* inoculation at 24 hpi; downregulated genes (black) and upregulated genes (red). **(C)** Comparison of gene expression values of RNA-Seq and qRT-PCR: correlation of fold change values for eight *F. vesca* genes obtained by RNA-Seq and qRT-PCR.

The transcriptome of white and red woodland strawberry fruits was analyzed in three biological replicates at 24 hpi. Twenty-four hours postinoculation was chosen as sampling time since the qPCR analysis showed that, at this point, the pathogen growth becomes active, especially in red fruits (**Figure 1B**). The 24 hpi was also reported previously as the most transcriptionally responsive time point in *F.* × *ananassa* strawberry ripe fruits in response to *B. cinerea* (Xiong et al., 2018). The number of reads mapped on *F. vesca* genome is provided in **Supplemental Table 2**. Based on principal component analysis, samples were separated by their infection state along the second component and by their ripening stage along the first component (**Figure 2A**).

The DEGs between *Botrytis*- versus mock-inoculated samples, in both white and in red fruits, were computed. Accordingly, in white fruits, the DEGs were 1,303, out of which 588 were upregulated, whereas in red fruits, 649 of the 987 DEGs were upregulated (**Figure 2B**; **Supplemental Table 4**). The fold-change values from RNA-Seq analysis were validated using quantitative real-time PCR (qRT-PCR) assay. The expression measurement of eight *F. vesca* genes (**Supplemental Table 3**) performed by qRT-PCR showed similar expression pattern to those of the RNA-Seq values (R^2^ > 0.88; **Figure 2C**).

DEGs were functionally categorized using MapMan (**Supplemental Table 5**). Among the total DEGs, only 149 were common to both growth stages (**Supplemental Table 3**). These common genes regulate a number of functions (**Supplemental Figure 3**). Interestingly, the common genes in protein and RNA modifications (pentatricopeptide repeat-containing proteins genes of the “Defense/Stress” class and genes of “RNA processing” classes) and biotic stress (genes of “Recognition and Signaling” class) functional classes were mostly downregulated in white fruits (**Supplemental Figure 3**).

With regard to the *Botrytis* transcriptome, the number of reads mapped on *Botrytis* genome from the white or red inoculated fruits was very low (**Supplemental Table 2**), prohibiting an in-depth analysis of fungal transcriptional activity during early infection.

### Biological Responses of *F. vesca* Strawberry Fruits to *B. cinerea* Inoculation

**Figure 3:**
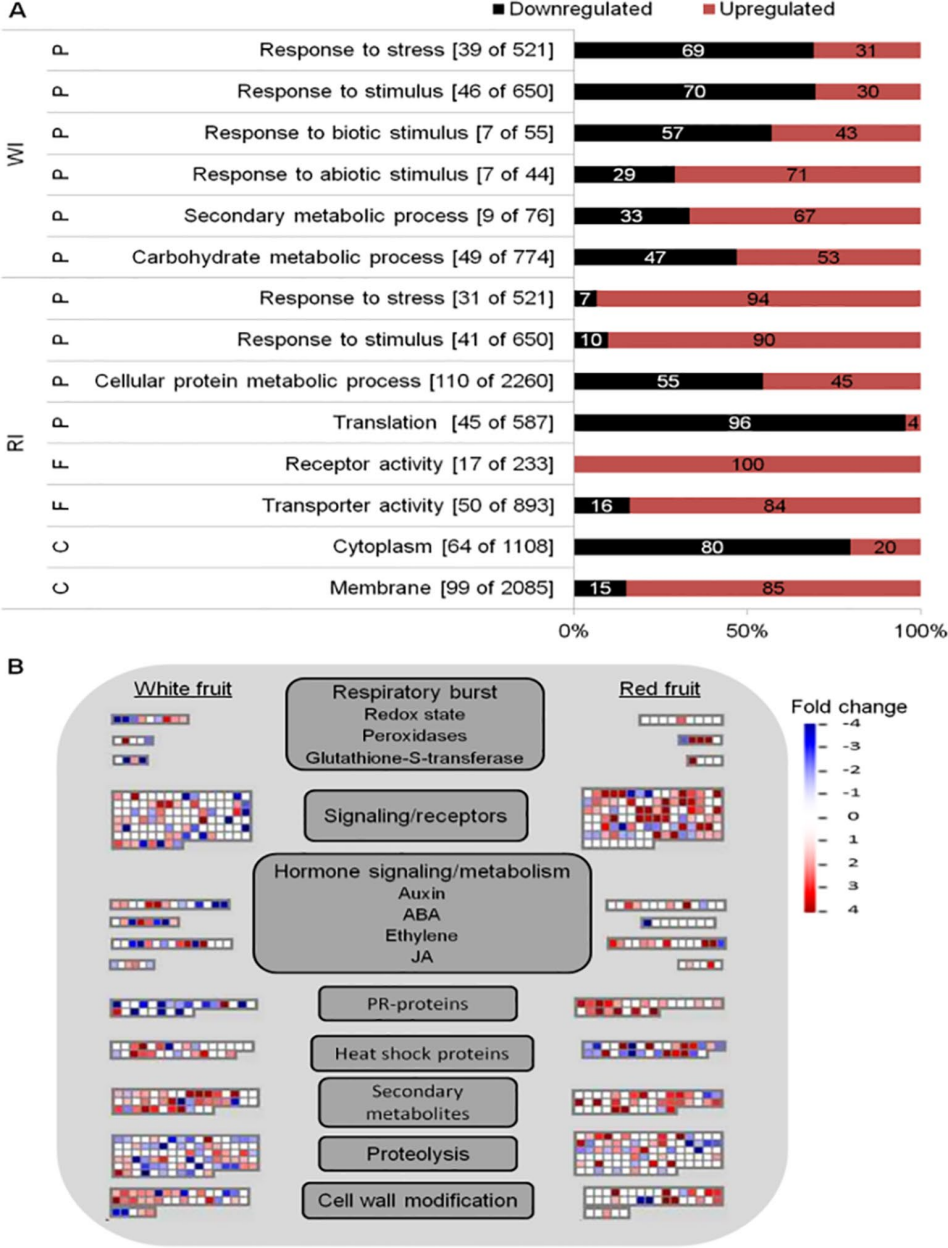
Functional enrichment and biotic stress overview of the differentially expressed (DE) genes of *Fragaria vesca* in *Botrytis cinerea* inoculated white and red fruits, 24 h postinoculation. **(A)** Functionally enriched classes in the DE genes of white inoculated (WI) and red inoculated (RI) fruits, using AgriGO analysis tool. The number of DE genes out of the total *F. vesca* genes in each category is shown in parenthesis. The proportion of down- and upregulated genes within a category is represented by black and red bars, respectively. C, cellular component; F, molecular function; P, biological process. **(B)** Overview of biotic stress changes in infected fruits, visualized by MapMan. Up- and downregulated genes are shown in red and blue, respectively. The scale bar displays fold change values; fold change value “0” is given for genes which are not DE. ABA, abscisic acid; JA, jasmonic acid.

Global analyses of infected fruits DEGs were computed to have an overview of transcriptional regulation in response to *Botrytis* infection (**Figure 3**). Enrichment of GO terms was evaluated to know the biological processes, molecular functions, and cellular components mostly affected by *B. cinerea* infection. From the GO terms corresponding to biological processes, “response to stress” and “response to stimulus” were overrepresented in both white and red fruits; additional overrepresented GO terms in white fruit were those related to “responses to biotic stimulus,” “response to abiotic stimulus,” and “carbohydrate metabolic process,” whereas in ripe fruits, the “cellular protein metabolic process” functional class was the most represented one (**Figure 3A**; **Supplemental Tables 6**, **7**). As depicted in **Figure 3A**, differently from ripe fruits, in white fruits, downregulated genes dominated “response to stimulus” and “response to stress” GO terms, and among the genes those involved in hypersensitive response, such as TMV N resistance (Whitham et al., 1994; Marathe et al., 2002), were prominent (**Supplemental Table 7**). On the other hand, in red fruits, besides the category “response to stimulus,” upregulated genes dominated functional categories linked to pathogen defense, such as “receptor activity,” “transporter activity,” and “membrane” (**Figure 3A**).

The biotic stress pathway of the infected fruits was visualized *via* the MapMan tool (Thimm et al., 2004). Comparing the white with the red fruit transcriptomic response, a clear difference in biotic stress gene response is apparent, in both expression level and identity of genes with functions varying from recognition to defense responses to the pathogen (**Figure 3B**). Moreover, the number of differentially regulated genes (mainly upregulated) in “secondary metabolism” and “cell wall modification” was higher in white than in red fruits, but the opposite was true for signal transduction and proteolysis (**Figure 3B**). In addition,the defense response appeared stronger at the ripening stage, as PR coding genes were more represented and upregulated in red than white fruits (**Figure 3B**). However, it should be noted that the genes categorized as PR proteins encoding are TMV N proteins (**Supplemental Table 5**), according to the MapMan annotation used—*F. vesca* (Fvesca_226) mapping. However, these TMV N proteins are not considered members of the PR protein family (van Loon et al., 2006; Sudisha et al., 2012; Sinha et al., 2014).

Besides regulating developmental processes, phytohormones are involved in plant defense-signaling pathways (Bari and Jones, 2009). As expected, the involvement of hormonal signaling in *Botrytis*–strawberry fruit interaction was shown in the MapMan visualization. The involvement of auxin, ABA, ethylene (ET), and jasmonic acid (JA) phytohormones was highlighted (**Figure 3B**). Genes involved in their biosynthesis or signaling, such as 1-aminocyclopropane-1-carboxylate (ACC) oxidase, ethylene response transcription factors, allene oxide cyclase (AOC), 12-oxophytodienoate (OPDA) reductase, lipoxygenase, 9-cis-epoxycarotenoid dioxygenase (NCED), and zeaxanthin epoxidase (ZEP) were differentially regulated (**Supplemental Table 5**). Auxin, ABA, ET, and JA have been reported to play a central role in plant defense against *B. cinerea* (Audenaert et al., 2002; El Oirdi et al., 2011; Osorio et al., 2011).

### Pathogen Perception and Signal Transduction Genes Are Modulated

Recognition is one of the earliest events in plant–pathogen interaction, before triggering other cascades of the defense response. Membrane-localized receptor-like kinases (RLKs) mediate cell wall–plasma membrane cross-communication, helping plants to respond to various stimuli, an essential prerequisite for pathogen recognition and activation of defense responses (Brutus et al., 2010; Singh and Zimmerli, 2013; Burdiak et al., 2015). In total, about 60 membrane-localized *RLK* genes were differentially expressed in white and red fruits; only three RLK genes (*17154*, *22229*, and *24962*) were common for both fruit growth stages (**Table 1** and **Supplemental Table 8**). More than 70% of the differentially expressed RLK genes encode for proteins containing a leucine-rich repeat domain. The other RLK genes comprised cysteine-rich receptor kinases, lectin receptor kinases, and wall-associated receptor kinases (WAK) domains. Almost all RLK genes were upregulated in red fruits and only half of them in the white ones. It is known that repression of RLK genes that negatively regulate plant basal defense could lead to a reinforcement of the defense response (Delteil et al., 2016). Likewise, in the white fruit, *Cysteine-rich RLK28* and *Cysteine-rich RLK29* genes (*17514* and *21516*), involved in cell-death-mediated resistance (Yadeta et al., 2017), were downregulated (**Table 1**). Unexpectedly, RLK genes involved in immune response to necrotrophs, such as *Somatic embryogenesis receptor kinases* (*16992* and *26498*) and *ERECTA* genes (*19894*) (Godiard et al., 2003; Llorente et al., 2005; Heese et al., 2007), were repressed (**Table 1**). With regard to *WAK* genes, *WAK9* and *WAK14* in red fruits (*34321* and *15062*) and *WAK4* in white fruits (*34339*) were upregulated (Table 1). Although reports on the involvement of *WAK4* in plant defense are scanty (Lally et al., 2001), in white fruit, its induction was the highest (fourfold) among the RLKs. WAKs are known to serve as a sensor for monitoring cell wall integrity (Kohorn, 2015).

**Table 1:** Selected *Botrytis*-induced genes in *Fragaria vesca* fruits at 24 hpi with *P* ≤ 0.05 and absolute fold change (log2) of ≥0.8.

Gene ID	Annotation	White (24 hpi)		Red (24 hpi)	
		Fold change (log2)	*P* value	Fold change (log2)	*P* value
**Recognition and signaling**					
gene09392	Calmodulin 27	0.9	3.86E−02	−1.2	5.95E−03
gene21516	Cysteine-rich RLK28	−2.3	1.27E−02		
gene17154	Cysteine-rich RLK29	−4.4	1.85E−04	1.9	3.61E−02
gene04409	Disease resistance protein RGA3	−1.0	2.58E−03		
gene18440	Disease resistance protein RGA3			1.8	1.30E−02
gene29796	Glutamate receptor	2.4	2.95E−02	2.2	2.15E−02
gene24962	Lectin containing receptor kinase A4.3	1.3	4.08E−02	2.4	1.62E−02
gene24085	Mitogen-activated protein kinase	1.8	2.33E−02	1.3	4.16E−02
gene22229	Protein STRUBBELIG-RECEPTOR FAMILY 2	1.5	1.40E−02	2.9	9.37E−04
gene19894	RLK- ERECTA	−2.2	4.15E−02		
gene16992	Somatic embryogenesis receptor kinase 1	−2.6	1.71E−02		
gene26498	BRI 1-associated receptor kinase 1	−2.8	7.73E−03		
gene03058	TMV resistance protein N	−2.6	2.53E−02	2.4	3.77E−02
gene24787	TMV resistance protein N	−2.5	1.96E−02		
gene14188	TMV resistance protein N			2.3	7.86E−03
gene15062	Wall-associated receptor kinase-like 14			0.8	1.72E−02
gene34339	Wall-associated receptor kinase-like 4	2.1	1.00E−02		
gene34321	Wall-associated receptor kinase-like 9			0.9	1.64E−02
**Transcription factors**					
gene08006	Transcription factor MYB98	2.5	2.41E−02		
gene03712	Transcription factor MYB21			0.9	1.71E−02
gene24577	Transcription factor bHLH95	1.2	4.81E−03	1.6	1.43E−03
gene01197	Probable WRKY transcription factor 2	0.8	4.23E−02		
gene17698	Ethylene-responsive transcription factor	2.2	1.54E−02		
**Response to stress and secondary metabolism**					
gene13673	4-coumarate-CoA ligase	2.4	4.26E−02		
gene09753	Phenylalanine ammonia-lyase	1.5	4.23E−03	1.7	2.33E−03
gene12577	4-coumarate-CoA ligase			1.3	1.92E−02
gene23367	Chalcone-flavonone isomerase	1.3	8.82E−04	1.5	5.09E−04
gene26826	Chalcone synthase	1.3	2.10E−03	1.5	9.79E−04
gene03747	Dihydroflavonol-4-reductase	−1.7	3.57E−02		
gene15176	Bifunctional dihydroflavonol 4-reductase			1.6	3.04E−02
gene28142	Cationic peroxidase 1	2.8	4.99E−02	3.2	1.30E−02
gene10758	Peroxidase	−1.3	2.23E−02		
gene00894	Lignin-forming anionic peroxidase			2.3	1.82E−02
gene24296	Laccase	2.7	2.07E−02		
gene12086	Laccase	1.6	4.29E−03	2.0	6.24E−03
gene15030	Chitotriosidase-1	3.5	1.00E−02		
gene14601	Thaumatin	1.3	3.84E−03		
gene32423	Osmotin-like protein	1.2	1.43E−02		
gene30565	Pathogenesis-related protein PR-4B			1.9	2.20E−02
gene32087	Endoglucanase 6	1.2	8.44E−03		
gene20640	Beta-glucosidase 12	-2.8	6.67E−03		
gene23960	Dirigent protein	6.1	4.53E−02		
gene06528	Beta-galactosidase 9	-2.1	1.47E−02	1.2	2.87E−02
gene07064	Major allergen Pru ar 1/PR Bet v	2.5	3.53E−03	1.5	1.52E−02
gene05122	Major allergen Pru ar 1/PR Bet v	2.2	3.03E−03		
gene00814	Defensin-like protein	−1.1	3.03E−03	−1.0	2.23E−03
**Cell wall**					
gene20547	Cellulose synthase-like protein	1.2	8.50E−03	2.1	2.52E−04
gene19766	Long-chain-alcohol O-fatty-acyltransferase 5	1.9	2.33E−02	1.2	1.71E−02
gene24409	Callose synthase 9	-2.1	2.77E−03	2.33	5.22E−03
gene13718	Xyloglucan endotransglucosylase	1.3	2.81E−02		
gene00661	Xyloglucan endotransglucosylase			−2.3	3.57E−03
gene30789	Polygalacturonase	−1.3	6.33E−03	1.7	5.47E−03
gene21638	Polygalacturonase	1.5	3.62E−02		
gene02221	Expansin-A4	1.7	1.34E−02		
gene24370	Pectate lyase 1	0.9	3.09E−02	0.9	3.41E−02
gene23429	Pectate lyase 12	−1.8	1.33E−02		
gene17879	Pectinesterase 51	0.8	3.70E−02		
gene31599	Pectinesterase 29	−1.2	1.15E−02		
gene10629	Pectinesterase 41			2.9	2.89E−02

The infection of *Botrytis* affected the regulation of 25 genes putatively encoding for heat shock proteins (HSP) (**Supplemental Table 8**). HSPs can act as molecular chaperones to control functionality of plasma-membrane-resident receptors and intracellular resistance (R) proteins against pathogens (for review, see Park and Seo, 2015). In *F.* × *ananassa* strawberry leaves, HSP showed a high response to *Colletotrichum fragariae* infection (Fang et al., 2012). In our case, more than 10 HSP genes were upregulated, and in the red fruit, the upregulation level of some of these HSP genes was among the highest: about 34-fold increase for *07771* gene and 23-fold for *20877* gene (**Supplemental Table 8**).

The signal from cell membrane is downstreamed to cytoplasm *via* serine/threonine-protein kinase receptor (SRK) and further relayed by mitogen-activated protein kinases (MAPKs) and calcium-dependent protein kinase (CDPK) for transcriptional reprogramming needed in defense (Afzal et al., 2008; Boudsocq et al., 2010). No SRK or CDPK gene was transcriptionally altered in white fruits, but there were three (*30638*, *30648*, and *30753*) and two (*17503* and *18254*) genes, respectively, upregulated in red fruits (**Supplemental Table 8**), suggesting that in white fruits, downstream signal transduction between membrane-bound receptor kinases and MAPKs was regulated. Of the eight MAPKs DEG, only gene *24085* was common to both fruit-ripening stages, and unlike in white fruits, in red fruits, all of the MAPKs were highly upregulated (**Supplemental Table 8**). This might be due to the much higher disease pressure on red than white fruits, as can be seen in **Figure 1**. Some of these genes were found differentially regulated at 24 hpi in susceptible *F.* × *ananassa* ripe fruit inoculated with *B. cinerea* (Xiong et al., 2018), suggesting the existence of a similar signaling cascade to transduce immune signaling in ripe woodland strawberry upon *B. cinerea* infection.

On the other hand, surprisingly, *Botrytis* infection stimulated the expression of a number of R genes with a nucleotide binding site leucine-rich repeat (NBS–LRR) and of the pentatricopeptide (PPR) repeat-containing protein types, both in white and red fruits (**Table 1** and **Supplemental Table 5**). This was quite novel because, with the exception of a few R proteins such as *Arabidopsis* resistance to *Leptosphaeria maculans* 3 (RLM3) and wheat TaRcR1, known to be associated to necrotroph pathogens (Staal et al., 2008; Zhu et al., 2017), R proteins in general are not associated with a defense response to necrotrophs. On the contrary, in the interaction of host with many biotrophic and hemibiotrophic pathogens, the pathogen effectors cause the induction of R genes to mediate effector-triggered host immunity (Jones and Dangl, 2006). However, in some interactions with necrotrophs, the toxins produced by the pathogen induce R genes to promote infection (Lorang et al., 2007; Wang et al., 2007; Faris et al., 2010). R-gene-mediated susceptibility to necrotrophs is envisaged as ectopic expression of hypersensitive response, which could allow the pathogen to access an initial growth substrate (Laluk and Mengiste, 2010; Mengiste, 2012).

PPR proteins are largely known for their role in modulating RNA-binding proteins, which mediates the expression of genes involved in photosynthesis, respiration, plant development, and environmental responses (Nakamura et al., 2012; Barkan and Small, 2014). There are also many PPR proteins that have been identified to function in defense against necrotrophic pathogens (Laluk et al., 2011; Xing et al., 2018). In this study, 47 genes putatively annotated for PPR proteins were differentially regulated (**Supplemental Table 5**), suggesting their involvement in *F. vesca* strawberry–*Botrytis* interaction. With regard to NBS–LRR, 45 genes putatively annotated for TMV N resistance, recognition of *Peronspora parasitica* (RPP), and resistance gene analogues (RGAs) were transcriptionally altered both in white and red fruits (**Table 1** and **Supplemental Table 8**). Almost all of these NBS–LRR genes were upregulated in red fruits and downregulated in white ones. In particular, the members of the TMV N gene family were largely represented with several members differently distributed in the two transcriptomes. Of these genes, *03058*, *31994*, and *16534* were shared by the two ripening stages, but like all the others members, they were oppositely regulated by the pathogen infection (**Supplemental Table 8**).

TMV N and RPP genes are known to be involved in a hypersensitive response at the site of pathogen entry (Whitham et al., 1994; Cooley et al., 2000; Marathe et al., 2002; Takahashi et al., 2002; Bent and Mackey, 2007). It is also known that the hypersensitive response facilitates the colonization of necrotroph pathogens such as *B. cinerea* (Govrin and Levine, 2000). Studies show that *B. cinerea* elicits the expression of hypersensitivity-related genes in its hosts to facilitate infection (Govrin et al., 2006; Rossi et al., 2011); however, with regard to TMV, RPP, and RGA genes, there is no report that the fungus manipulates these genes to establish infection. Nonetheless, their upregulation in susceptible ripe fruits and suppression in unripe white fruits suggest that they might assist *Botrytis* colonization of the ripe fruits.

In addition, TMV N components of the NBS–LRR repertoire are known to function through signaling cascades that involve ubiquitination for protein degradation (Marathe et al., 2002).

The functional category “protein degradation” was one of the most represented in the DEGs: 61 in white and 42 in red fruits; and about one-third of which are ubiquitin genes where E3 ubiquitin ligases dominated (**Figure 3B** and **Supplemental Table 5**). Ubiquitins are a diverse family of proteins involved in posttranslation modification with different biological roles, including plant immunity (Zeng et al., 2006; Cheng and Li, 2012; Li et al., 2016). Many of these ubiquitin ligases have not yet been characterized in *Fragaria* species. However, it is known that E3 ubiquitin ligases are associated with pathogen and abiotic stress responses (Luo et al., 2010; Zhou and Zeng, 2017). The intricate regulatory network of E3 ligases might be involved in determining the different response of strawberry white and red fruits. Further studies are needed to clarify their regulatory mechanisms influencing defense pathways and immunity responses during strawberry–*Botrytis* interaction.

### Phytohormone Biosynthesis and Metabolism

Genes involved in the biosynthesis and metabolism of phytohormones, except salicylic acid (SA), were differentially regulated, following *B. cinerea* inoculation (**Supplemental Table 5**). According to the putative functions of the *F. vesca* DEGs, *ACC-oxidase*, *AOC*, *OPDA-reductase*, and *lipoxygenase* genes involved in the important steps of ET and JA biosynthesis were upregulated at both fruit ripening stages. *F.* × *ananassa*, achenes of red strawberry fruits were reported to produce ET at a low concentration (Iannetta et al., 2006), while fruits were found responsive to ET when applied externally (Villarreal et al., 2010). In these studies, fruits that received ET externally were prone to *B. cinerea*. In this regard, the pathogen itself could trigger the production of ET in host tissue. A transcriptomic study on tomato suggested that *B. cinerea* would induce the expression of genes involved in ripening to favor its development (Cantu et al., 2009). ET and JA are usually associated with defense against necrotrophic pathogens, but their pathways can also functionally interact with SA signaling pathway to fine-tune plant defense (Kunkel and Brooks, 2002; Beckers and Spoel, 2006). However, no SA marker gene was differentially modulated in both ripening stages.

Apart from ET and JA, *Botrytis* infection affected the expression level of genes involved in the biosynthesis and metabolism of auxin, ABA, and gibberrelic acid, more in white fruits than in red ones (**Supplemental Table 5**). Auxins have mainly been implicated as key regulators for growth and fruit ripening in strawberry (Aharoni et al., 2002; Mezzetti et al., 2004); however, there is evidence that this growth regulator is also involved in the strawberry defense response (Osorio et al., 2011). Most related DEGs that encode auxin-induced and responsive proteins were downregulated in the resistant white fruit (as low as −42-fold change; **Supplemental Table 5**). Consistent to this, decrease in auxin content as well as enhanced expression of some auxin-repressed genes in fruit of transgenic *F. vesca* FaPE1 lines were found to be correlated with *B. cinerea* resistance (Osorio et al., 2011). ABA also showed a stronger response to infection in white fruits compared to red fruits (**Supplemental Table 5**). According to the RNA-Seq data, *NCED1* and *ZEP* genes involved in ABA biosynthesis were upregulated but only in white fruit (**Supplemental Table 5**). ABA is known to promote strawberry ripening, also through the upregulation of *WRKY* genes (Jia et al., 2011; Li et al., 2019), and to be involved in defense responses to pathogens, where its deficiency made tomato and *Arabidopsis* more resistant to *B. cinerea* through its effects on reactive oxygen intermediates and cell wall stiffening (Audenaert et al., 2002; AbuQamar et al., 2006; Asselbergh et al., 2007; Curvers et al., 2010). Moreover, in *F.* × *ananassa* strawberry, different WRKY transcription factors were recently shown to be upregulated by ABA. Here, the transcription level of *FaNCED1* genes were congruent with fruit ABA content, promoting ripening (Jia et al., 2011). Interestingly, two NECD1-encoding genes (*31335* and *30616*), which could be taken as a “susceptible factor,” were upregulated in the resistant white fruits only. It is known that *B. cinerea* synthesizes plant hormone analogues and elicitors (Chagué et al., 2002; Siewers et al., 2004; Siewers et al., 2006) able to induce hormone biosynthesis pathways in order to affect the host’s susceptibility by altering the hormone balance in infected host tissues (Sharon et al., 2004; El Oirdi et al., 2011).

Overall, such complex interplay among the phytohormones was also observed during *B. cinerea* infection of grapevine inflorescence as well as ripe and unripe tomato fruits (Blanco-Ulate et al., 2013; Haile et al., 2017). This suggests that modulation of phytohormones plays a role in regulating the interaction between strawberry fruit and *B. cinerea*, most likely in a ripening-stage specific manner. Nevertheless, it is difficult to conclude whether the combined changes in phytohormone-related processes promoted or inhibited immunity in *F. vesca* since much of the DEGs are involved in the biosynthesis pathway.

### Defense-Related Genes of White and Red Fruits Are Differentially Regulated

Following recognition of *Botrytis* infection and queued signaling cascades, the expression level of a number of defense-related genes was altered both in white and red fruits, including the related transcription factors (TF) (**Supplemental Table 5**). In this experiment, the expression levels of more than 100 genes putatively encoding TFs belonging to different families were modulated in both white and red fruits (**Table 1** and **Supplemental Table 9**). Besides TFs regulating hormone biosynthesis, bHLH, MYB, WRKY, and zinc fingers TF domains were the predominant ones. TFs play a role in biotic stress by regulating genes involved in innate immunity, hormone signaling pathways, and phytoalexin synthesis (Mzid et al., 2007; Gao et al., 2011; Höll et al., 2013). For example, ectopic expression of grapevine VvWRKY2 in tobacco enhances resistance to necrotrophic fungi such as *B. cinerea* (Mzid et al., 2007), and MYB98 regulates the activation of genes required for defense (Punwani et al., 2008). In line with this, genes putatively annotated for these two TFs (*01197* for WRKY2 and *08006* for MYB98) were upregulated only in white fruit.

With respect to pathogenesis-related proteins (PRs), 28 putative PR genes were differentially regulated, mainly in white fruits as shown in **Supplemental Table 10**. Additionally, out of the 17 categories of PR protein families (van Loon et al., 2006; Sudisha et al., 2012; Sinha et al., 2014), genes belonging to 9 of them were transcriptionally altered. For genes like chitinase (*15030*), the upregulation was more than 10-fold in white fruits. In the case of β-1,3-glucanase, two genes in white fruits and one in red fruits were upregulated following *Botrytis* infection. Glucanases and chitinases are the most abundant classes of strawberry PR genes having hydrolytic activity (reviewed in Amil-Ruiz et al., 2011). Moreover, osmotin and thaumatin PRs were also induced in white fruits only. These proteins belong to PR-5 protein family, well known to inhibit fungal growth including *B. cinerea* (Monteiro et al., 2003; González et al., 2017). Notably, we found defensin and lipid transfer proteins (belonging to PR-12 and 14 protein family, in respective order), functioning as antimicrobial peptides (Garcia-Olmedo et al., 1995; Ganz, 2003; Nanni et al., 2014), downregulated in white fruits.

Other PR proteins whose expression was altered due to *Botrytis* infection were those homolog to the major allergen pru ar 1 protein, a PR-10 protein. Among five upregulated genes encoding for major allergens pru ar 1 proteins (**Supplemental Table 10**), only one gene (*07064*) was common to white and red fruits, while the others were upregulated in white fruits. PR-10 proteins are known to be induced in *F.* × *ananassa* strawberry fruits challenged by *B. cinerea* and *Colletotrichum acutatum* (Guidarelli et al., 2011; Xiong et al., 2018) as well as in leaves infected with *Podosphaera aphanis* (Jambagi and Dunwell, 2015) and in roots infected with *Phytophthora cactorum* (Toljamo et al., 2016). These proteins are important for defense against pathogens in strawberry, and they are expressed faster and/or stronger in resistant genotypes or resistant fruit phenological stages (unripe ones) compared to susceptible genotypes or susceptible fruit stages (Guidarelli et al., 2011; González et al., 2013). PR-10 proteins bind to different biologically important ligands, including sterols and flavonoids, and in strawberry, they were observed to interact with metabolic intermediates of flavonoid biosynthesis to regulate the biosynthesis pathway (Koistinen et al., 2005; Zubini et al., 2009; Muñoz et al., 2010; Casañal et al., 2013). This entails that the upregulation of genes encoding these proteins in response to *Botrytis* infection might occur to enhance flavonoid biosynthesis. The expression profiles of TFs and PR-protein genes suggest that defense against *B. cinerea* infection was elicited earlier in white fruits.

### Secondary Metabolites Biosyntheses Pathways Are Triggered in Infected Fruits

In response to *Botrytis* infection, a number of genes involved in the synthesis of secondary metabolites, in particular those in flavonoid biosynthesis, were differentially regulated (**Supplemental Table 5**). Genes involved in terpenoid biosynthesis, according to KEGG Pathway database (Kanehisa and Goto, 2000), were also induced. In white fruits, three genes putatively encoding geranylgeranyl transferase, gibberellin 3-beta-dioxygenase, and ent-copalyl diphosphate synthase (*12147*, *14841*, and *20295*, respectively), which are involved in di- and tetra-terpenoid biosynthesis, were upregulated. Conversely, in red fruits, one gene (*12609*) putatively encoding (−)-germacrene D synthase, which catalyzes the first committed step in the synthesis of sesquiterpenoids, and two genes (*18138* and *31674*) putatively encoding carotenoid isomerase and phytoene synthase, respectively, involved in tetra-terpenoid biosynthesis, were upregulated. Isoprenoids appear to be part of the general defense responses in strawberry leaves and fruits, as genes involved in their biosynthesis were also induced during *Colletotrichum* spp. and *P. cactorum* infections (Hirai et al., 2000; Guidarelli et al., 2011; Toljamo et al., 2016).

**Figure 4:**
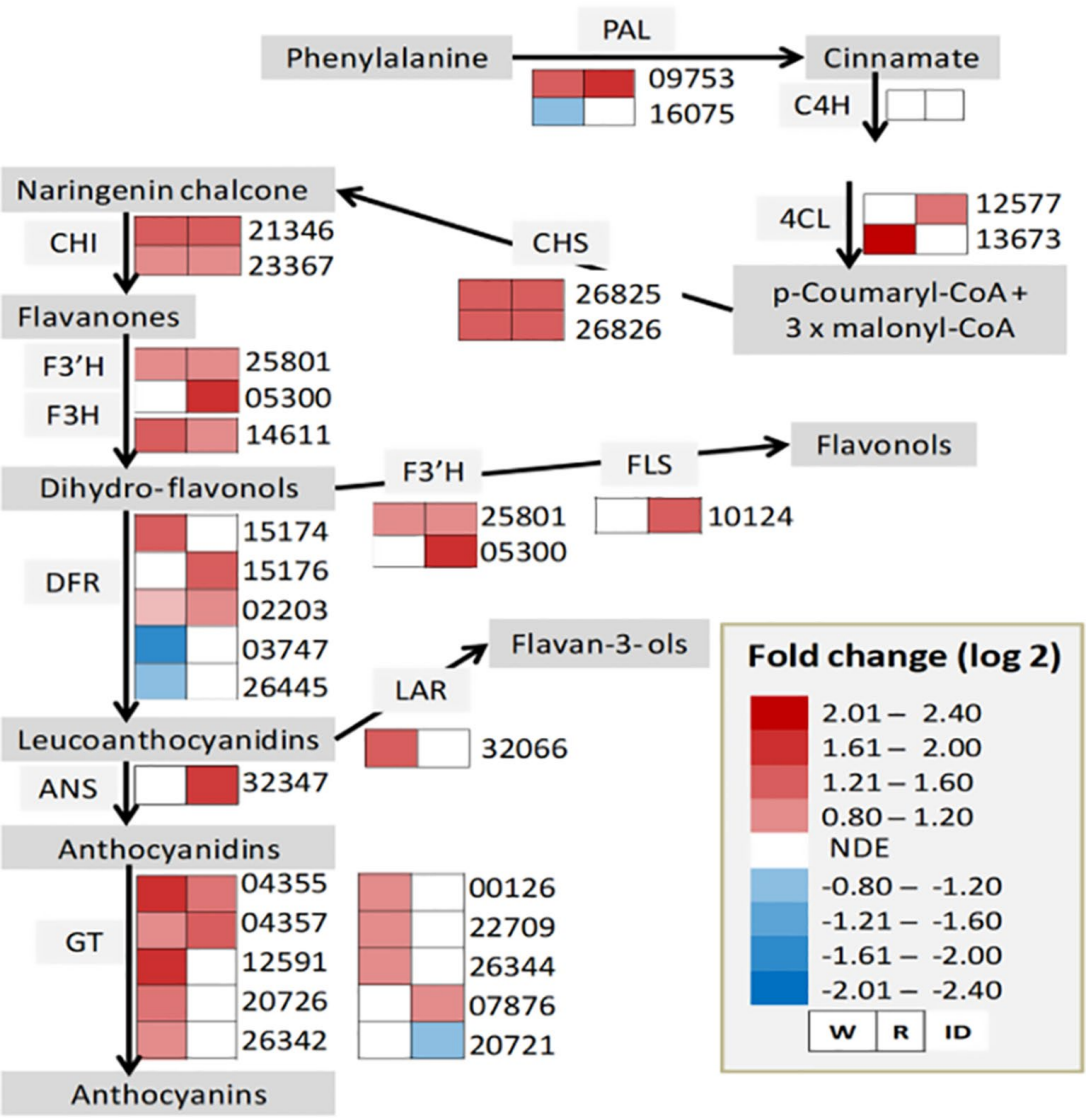
Flavonoid biosynthesis pathway in *Botrytis cinerea* inoculated *Fragaria vesca* fruits at 24 h postinoculation. Heatmaps of gene expression (from RNA-Seq result) in inoculated white (W) and red (R) fruits. ANS, anthocyanidin synthase; C4H, cinnamic acid 4-hydroxylase; CHI, chalcone isomerase; CHS, chalcone synthase; DFR, dihydroflavanol 4-reductase; F3’H, flavonoid 3’-monooxygenase; F3H, flavanone 3-hydroxylase; FLS, flavonol synthase GT, glycosyltransferase; LAR, leucoanthocyanidin reductase; PAL, phenylalanine ammonia-lyase; 4CL, 4-coumarate-coenzyme A ligase. NDE, not differentially expressed. Genes such as 02203, 05300, and 10124 are not in the DE genes list, but included for the completeness of the biosynthesis pathway. The expression profile for 02203 in W—log2 FC: 0.63, *P* value: 0.034; for 05300 in R—log2 FC: 2.0, *P*–value: 0.069; and for 10124 in R—log2 FC: 1.6, *P*–value: 0.053.

The upregulation of the major allergen pru ar 1 genes suggests the active involvement of a flavonoid biosynthesis pathway in the interaction between strawberry fruit and *B. cinerea*. As a result, the expression status of genes involved in the biosynthesis pathway was thoroughly investigated (**Figure 4**). As depicted in **Figure 4**, except for cinnamic acid 4-hydroxylase (C4H), genes encoding key enzymes required for flavonoid biosynthesis pathway were differentially altered, at least in one of the ripening stages, albeit on a different scale. Previous studies have shown that flavonoids play an important role in the defense response to pathogen attack. In grapevine flowers, flavonoid concentrations increased following *B. cinerea* inoculation as pathogen response mechanism (Haile et al., 2017). Similarly, in strawberry, an increased concentration of proanthocyanidins around the penetration site of *B. cinerea* on immature fruits was reported to keep the pathogen under quiescence (Jersch et al., 1989). Flavan-3-ol derived compounds also seem to be involved in pathogen resistance in strawberry (Yamamoto et al., 2000; Puhl and Treutter, 2008; Nagpala et al., 2016; Toljamo et al., 2016). When white and red *F.* × *ananassa* strawberry fruits were challenged with *B. cinerea* and *C. acutatum*, a relatively higher accumulation of flavan-3-ols (catechin, procyanidin B1, and procyanidin B3) was observed in less susceptible white fruits with respect to red ones (Nagpala et al., 2016). Interestingly, in our study, gene encoding leucoanthocyanidin reductase (LAR), which converts leucoanthocyanidins to flavan-3-ols, was upregulated only in white fruits (**Figure 4**). Since *Botrytis* progress in white fruit was inconspicuous (**Figure 1A**), flavonoid polyphenols could play a role in determining the susceptibility of strawberry fruit to *B. cinerea*.

### Cell Wall Modification of Strawberry Fruits Following *B. cinerea* Infection

The first obstacles that plant pathogens stumble upon are the cuticle and cell wall, the forefront of the plant defense system. Upon contact with a pathogen, the defense system undergoes changes to physically and chemically hinder the penetration *via* antimicrobial enzymes and secondary metabolites to interfere with the biology of the intruder (reviewed in Malinovsky et al., 2014). In the *Botrytis*-induced strawberry trancriptome, 56 genes involved in cell wall biosynthesis or modification were differentially regulated (**Table 1** and **Supplemental Table 11**). Endogenous cell wall modifying/degrading proteins were prominent, especially in white fruits. For example, genes encoding expansins, known to endogenously influence cell wall extensibility and necrotroph susceptibility (AbuQamar, 2014), were unexpectedly upregulated in white fruits (**Supplemental Table 11**). The upregulation of genes encoding enzymes endogenously degrading cell wall such as endoglucanase, rhamnogalacturonate lyase, xyloglucanas endotransglucosylase, polygalacturonase, pectate lyase, and pectinesterase (**Supplemental Table 11**) during pathogen attacks appears counterintuitive. Nevertheless, for most of the enzymes, downregulation of few redundant genes was also observed. For example, pectinesterases are involved both in cell wall loosening, by making polygalacturonans accessible to degradation by polygalacturonases, and in cell wall strengthening, by increasing the availability of polygalacturonan to Ca^2+^ binding (Micheli, 2001).Therefore, the induction and suppression of pectinesterases and polygalacturonases suggest that, in white fruits, cell wall stiffening and cell expansion and separation occur as simultaneous processes to counteract the pathogen growth while allowing for cell expansion, since the white fruit itself was also in its active growth phase. On the other hand, genes encoding glucosidase, which degrades cellulose and hemicellulose (Gilbert, 2010; Blanco-Ulate et al., 2014), and galactosidase, a ripening-related cell wall softening enzyme (Nunan et al., 2001), were switched off in infected white fruits.

From the gene expression profiles, processes as the oxidative burst mediated cross-linking of cell wall through germin-like proteins and extensin interaction (Bradley et al., 1992; Godfrey et al., 2007) and callose apposition are suppressed in white fruits, (**Supplemental Table 11**). On the other hand, both in white and red fruit transcriptome, an increased expression (up to fivefold) of genes involved in cutin and wax biosynthesis was found, as shown by the fivefold induction of gene *03099*, which encodes for protein wax 2, involved in cuticle membrane and wax production (Chen et al., 2003). Hemicelluloses and cellulose biosynthesis was also triggered as indicated by the upregulation of *06827*, *10606*, and *32285* genes and cellulose synthase genes, but mainly in white fruits. Similarly, genes encoding peroxidases and laccases which polymerize monolignols into lignin and are critical for cell wall fortification were also upregulated (**Supplemental Table 11**). In *Arabidopsis*, reduced cellulose synthesis leads to ectopic lignin synthesis (Cano-Delgado et al., 2003), implying a compensatory cell wall integrity maintenance mechanism when the integrity is perturbed (Hamann and Denness, 2011; Hamann, 2012). Probably, the suppressed callose synthesis observed in white fruits might be compensated by an enhanced lignin synthesis. In this regard, hemicelluloses are known to play a major role in cell wall toughening by interacting with cellulose and lignin (Scheller and Ulvskov, 2010) to challenge pathogen intrusion. Similarly, the deposition of lignin or lignin-like phenolic polymers in the cell wall has been implicated in plant defense, arresting advancing pathogens and/or limiting their progress (Menden et al., 2007; Miedes et al., 2014; Kelloniemi et al., 2015; Haile et al., 2017). Besides acting as a physical barrier to pathogen invasion, the process of lignin biosynthesis pathway, phenylpropanoid pathway, could also produce compounds with a defense role. Interestingly, the most upregulated (66-fold) gene in infected white fruit was a *dirigent protein* gene (*23960*) (**Table 1**). Dirigent proteins control the stereochemical coupling of monolignol radicals during the biosynthesis of lignans (Davin et al., 1997), which are polyphenols reported to have antipathogenic activity (Davin and Lewis, 1992; Akiyama et al., 2007; Li et al., 2017). Dirigent proteins are often found to be transcriptionally activated under biotic stress conditions (Borges et al., 2013; Thamil Arasan et al., 2013; Haile et al., 2017). Genes encoding dirigent proteins were found upregulated during *B. cinerea* infection of grapevine flowers. In this work, cell wall reinforcement was addressed as one of the possible mechanisms by which the grapevine flower arrests the advancement of *Botrytis* (Haile et al., 2017). The strong upregulation of dirigent protein and cell wall modification gene expression in white unripe strawberry fruit suggests a similar mechanism of *F. vesca* to arrest *B. cinerea* intrusion.

## Conclusion

The transcriptome analysis of strawberry fruits at different ripening stages provided a comprehensive picture of the response of white and red fruits to *B. cinerea* infection. Genes involved in defense response pathways, from perceiving to combating the pathogen, were differentially regulated within 24 hpi in both white and red fruits, albeit quantitatively different. Yet, *Botrytis* progress was visible on red fruits only after 48 hpi. Indeed, it has been shown that plant tissues can trigger defense mechanisms even when overwhelmed by pathogen attack (Kelloniemi et al., 2015; Nagpala et al., 2016; Xiong et al., 2018). Even though reprogramming of red fruit transcriptome towards defense was observed, it did not lead to a switch off of cell-death-causing membrane-localized *RLK* and *NBS–LRR* genes, such as *Cysteine-rich RLK29* and a number of *TMV N*, *RPP*, and *RGA* genes, which could play a role as susceptibility factors. On the other hand, in white fruits, the downregulation of these membrane-localized *RLK* and *NBS–LRR* susceptibility genes, the upregulation of chitinase, osmotin, and thaumatin PR protein genes and the triggering of the biosynthesis flavonoid polyphenols are likely correlated with the competence of the white fruit to block *B. cinerea* growth. With regard to fruit cell wall fortification, crucial to stop *Botrytis* growth, the RNA-Seq result suggests that, in white fruits responding to *B. cinerea* infection, hemicellulose-, cellulose-, and lignin-based fortification of the cell wall plays an important role. Nevertheless, callose deposition and oxidative burst mediated cross-linking of the cell wall based on germin-like proteins and extension, another important cell wall stiffening mechanism against *Botrytis* progress (Kelloniemi et al., 2015; Haile et al., 2017), appeared not to be involved in white fruits.

## Data Availability

The datasets generated for this study can be found in National Center for Biotechnology Information (NCBI), BioProject accession code PRJNA530684.

## Author Contributions

ZH wrote the manuscript and followed the research. EN-D performed experiments and wrote the first draft of the MS. EN-D is the first co-author together with ZH. MM, PS, and KE made all the bioinformatic analysis from raw RNA-seq to gene mapping, identification, and statistical analysis. LZ initiated the experimental work. CM helped with project supervision and with MS writing. EB supervised the project and MS writing and provided financial support.

## Funding

Institutional funding was received from ‘Alma Mater Studiorum Università degli studi diBologna’.

## Conflict of Interest Statement

The authors declare that the research was conducted in the absence of any commercial or financial relationships that could be construed as a potential conflict of interest.
