# Supplementary material for: Transcriptome Profiles of Strawberry (Fragaria vesca) Fruit Interacting With Botrytis cinerea at Different Ripening Stages
Source: Front Plant Sci. 2019 Sep 18;10:1131. doi: 10.3389/fpls.2019.01131 (PMC6759788; doi:10.3389/fpls.2019.01131)
Supplement: Supplementary file 1 [file DataSheet_1.pdf]

Supplemental Figures

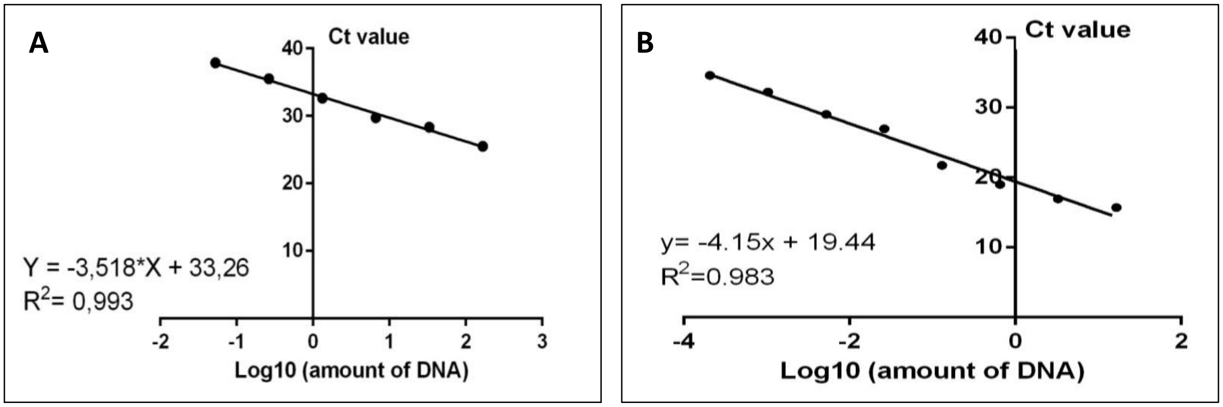

Supplemental Figure 1. Standard curves used for *Fragaria vesca* and *Botrytis cinerea* genomic DNA quantification. Curves were generated by amplifying the *EFTwo* (elongation factor 2-like) gene of *F. vesca* **(A)** and the *Bc3* (ribosomal IGS spacer) gene of *B. cinerea* **(B)**, in a 5-fold serially diluted genomic DNA of *F. vesca* and *B. cinerea*, respectively. The standard curves show good linear relationship (R2 > 0.98) between the log5 value of the starting DNA concentration and the threshold cycle (Ct), both in the plant and in the fungus.

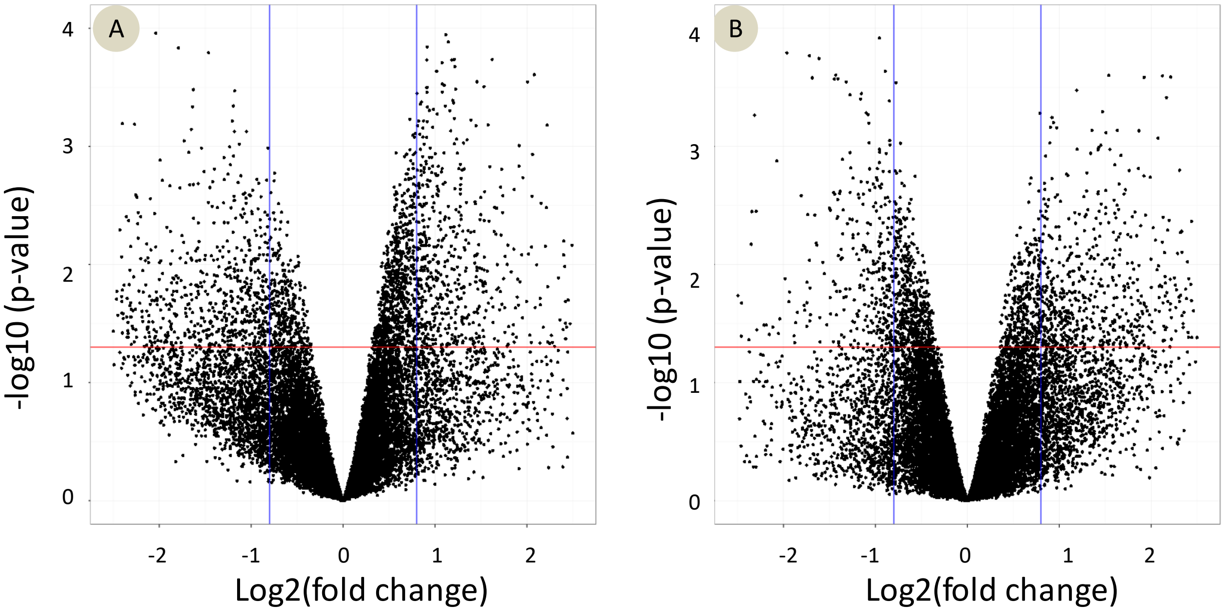

Supplemental Figure 2. Volcano plot of *Fragaria vesca* white fruit **(A)** and red fruit **(B)** gene expression values at 24 hpi. Genes in the left top rectangle [*P*. value > -log(10, 1.12) and fold change < log (2, |-0.80|)] were selected as downregulated; whereas, genes in the right top rectangle were selected as upregulated [*P*. value > -log(10, 1.12) and fold change > log (2, 0.80)].

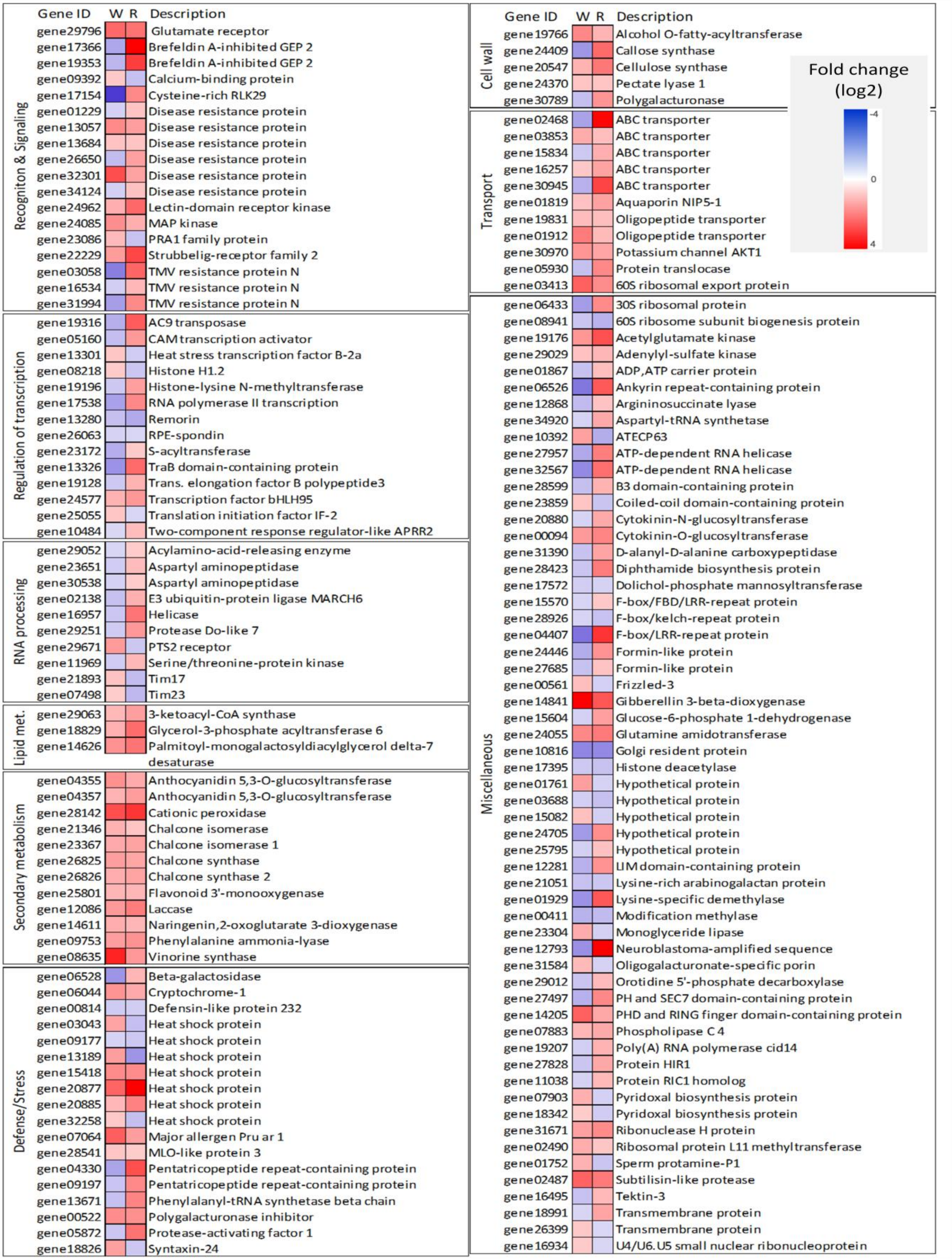

Gene ID W R Description
Recogniton & Signaling
gene29796 Glutamate receptor
gene17366 Brefeldin A-inhibited GEP 2
gene19353 Brefeldin A-inhibited GEP 2
gene09392 Calcium-binding protein
gene17154 Cysteine-rich RLK29
gene01229 Disease resistance protein
gene13057 Disease resistance protein
gene13684 Disease resistance protein
gene26650 Disease resistance protein
gene32301 Disease resistance protein
gene34124 Disease resistance protein
gene24962 Lectin-domain receptor kinase
gene24085 MAP kinase
gene23086 PRA1 family protein
gene22229 Strubbelig-receptor family 2
gene03058 TMV resistance protein N
gene16534 TMV resistance protein N
gene31994 TMV resistance protein N
Regulation of transcription
gene19316 AC9 transposase
gene05160 CAM transcription activator
gene13301 Heat stress transcription factor B-2a
gene08218 Histone H1.2
gene19196 Histone-lysine N-methyltransferase
gene17538 RNA polymerase II transcription
gene13280 Remorin
gene26063 RPE-spondin
gene23172 S-acyltransferase
gene13326 TraB domain-containing protein
gene19128 Trans. elongation factor B polypeptide3
gene24577 Transcription factor bHLH95
gene25055 Translation initiation factor IF-2
gene10484 Two-component response regulator-like APRR2
RNA processing
gene29052 Acylamino-acid-releasing enzyme
gene23651 Aspartyl aminopeptidase
gene30538 Aspartyl aminopeptidase
gene02138 E3 ubiquitin-protein ligase MARCH6
gene16957 Helicase
gene29251 Protease Do-like 7
gene29671 PTS2 receptor
gene11969 Serine/threonine-protein kinase
gene21893 Tim17
gene07498 Tim23
Lipid met.
gene29063 3-ketoacyl-CoA synthase
gene18829 Glycerol-3-phosphate acyltransferase 6
gene14626 Palmitoyl-monogalactosyldiacylglycerol delta-7 desaturase
Secondary metabolism
gene04355 Anthocyanidin 5,3-O-glucosyltransferase
gene04357 Anthocyanidin 5,3-O-glucosyltransferase
gene28142 Cationic peroxidase
gene21346 Chalcone isomerase
gene23367 Chalcone isomerase 1
gene26825 Chalcone synthase
gene26826 Chalcone synthase 2
gene25801 Flavonoid 3'-monooxygenase
gene12086 Laccase
gene14611 Naringenin,2-oxoglutarate 3-dioxygenase
gene09753 Phenylalanine ammonia-lyase
gene08635 Vinorine synthase
Defense/Stress
gene06528 Beta-galactosidase
gene06044 Cryptochrome-1
gene00814 Defensin-like protein 232
gene03043 Heat shock protein
gene09177 Heat shock protein
gene13189 Heat shock protein
gene15418 Heat shock protein
gene20877 Heat shock protein
gene20885 Heat shock protein
gene32258 Heat shock protein
gene07064 Major allergen Pru ar 1
gene28541 MLO-like protein 3
gene04330 Pentatricopeptide repeat-containing protein
gene09197 Pentatricopeptide repeat-containing protein
gene13671 Phenylalanyl-tRNA synthetase beta chain
gene00522 Polygalacturonase inhibitor
gene05872 Protease-activating factor 1
gene18826 Syntaxin-24
Gene ID W R Description
Cell wall
gene19766 Alcohol O-fatty-acyltransferase
gene24409 Callose synthase
gene20547 Cellulose synthase
gene24370 Pectate lyase 1
gene30789 Polygalacturonase
Fold change (log2)
-4
0
4
Transport
gene02468 ABC transporter
gene03853 ABC transporter
gene15834 ABC transporter
gene16257 ABC transporter
gene30945 ABC transporter
gene01819 Aquaporin NIP5-1
gene19831 Oligopeptide transporter
gene01912 Oligopeptide transporter
gene30970 Potassium channel AKT1
gene05930 Protein translocase
gene03413 60S ribosomal export protein
Miscellaneous
gene06433 30S ribosomal protein
gene08941 60S ribosome subunit biogenesis protein
gene19176 Acetylglutamate kinase
gene29029 Adenylyl-sulfate kinase
gene01867 ADP,ATP carrier protein
gene06526 Ankyrin repeat-containing protein
gene12868 Argininosuccinate lyase
gene34920 Aspartyl-tRNA synthetase
gene10392 ATECP63
gene27957 ATP-dependent RNA helicase
gene32567 ATP-dependent RNA helicase
gene28599 B3 domain-containing protein
gene23859 Coiled-coil domain-containing protein
gene20880 Cytokinin-N-glucosyltransferase
gene00094 Cytokinin-O-glucosyltransferase
gene31390 D-alanyl-D-alanine carboxypeptidase
gene28423 Diphthamide biosynthesis protein
gene17572 Dolichol-phosphate mannosyltransferase
gene15570 F-box/FBD/LRR-repeat protein
gene28926 F-box/kelch-repeat protein
gene04407 F-box/LRR-repeat protein
gene24446 Formin-like protein
gene27685 Formin-like protein
gene00561 Frizzled-3
gene14841 Gibberellin 3-beta-dioxygenase
gene15604 Glucose-6-phosphate 1-dehydrogenase
gene24055 Glutamine amidotransferase
gene10816 Golgi resident protein
gene17395 Histone deacetylase
gene01761 Hypothetical protein
gene03688 Hypothetical protein
gene15082 Hypothetical protein
gene24705 Hypothetical protein
gene25795 Hypothetical protein
gene12281 LIM domain-containing protein
gene21051 Lysine-rich arabinogalactan protein
gene01929 Lysine-specific demethylase
gene00411 Modification methylase
gene23304 Monoglyceride lipase
gene12793 Neuroblastoma-amplified sequence
gene31584 Oligogalacturonate-specific porin
gene29012 Orotidine 5'-phosphate decarboxylase
gene27497 PH and SEC7 domain-containing protein
gene14205 PHD and RING finger domain-containing protein
gene07883 Phospholipase C 4
gene19207 Poly(A) RNA polymerase cid14
gene27828 Protein HIR1
gene11038 Protein RIC1 homolog
gene07903 Pyridoxal biosynthesis protein
gene18342 Pyridoxal biosynthesis protein
gene31671 Ribonuclease H protein
gene02490 Ribosomal protein L11 methyltransferase
gene01752 Sperm protamine-P1
gene02487 Subtilisin-like protease
gene16495 Tektin-3
gene18991 Transmembrane protein
gene26399 Transmembrane protein
gene16934 U4/U6. U5 small nuclear ribonucleoprotein

Supplemental Figure 3. Heat map showing the expression profile of the 149 common genes. Gene description and functional category, based on MapMan annotation, is provided. W, white fruit; R, red fruit. The scale bar displays fold change values (log2).
