# Supplementary material for: Transcriptome Profiles of Strawberry (Fragaria vesca) Fruit Interacting With Botrytis cinerea at Different Ripening Stages
Source: Front Plant Sci. 2019 Sep 18;10:1131. doi: 10.3389/fpls.2019.01131 (PMC6759788; doi:10.3389/fpls.2019.01131)
Supplement: Supplementary file 2 [file DataSheet_2.pdf]

Supplemental Information 1. Modification to Porebsky et al. (1997) DNA extraction protocol, modifications are in red font.

One hundred milligrams of samples powder was transferred to a new 1.5 mL polypropylene tube using a sterile spatula. At this time, 1 mL of DNA extraction buffer [100mM Tris-HCl pH 8.0, 20 mM EDTA pH 8.0, 1.4M NaCl, 2 % w/v CTAB (hexadecyltrimethylammoniumbromide)]and 0.4ul of β-mercaptoethanol (0.4% were added. The mixture was vortexed for 1min, mixed by 2-3 inversion and then incubated for 2 hours at 65 °C in a water-bath. After addition of the powdered leaf material and immersion in the 65 °C water bath, the mixture became clear in a few seconds, as soon as the different reagents interacted with proteins, phenolic compounds and polysaccharides. After incubation, 1 mL of chloroform/octanol (chl/oct, 24:1) was added to the tube, the mixture was vortexed thoroughly until making an emulsion and centrifuged 5 min at 8000 rpm. The aqueous phase was transferred to a new 2 mL tube and RNA digestion was performed by adding 5ul of RNAse (1mg in $0.1ml^{-1}$) and incubating for 30min at 37°C. An equivalent volume of chl/oct was added. The tube was vortexed and centrifuged 5 min at 8000rpm. The upper phase was transferred in a new 2ml tube and 0.8 volume of isopropanol was added.
The tube was swirled gently and kept 2min at room temperature. After a white DNA precipitate appeared, the tube was then centrifuged 22 min at 14 000rpm and the supernatant was withdrawn. The DNA pellet was washed with 500ul 70 % ethanol, centrifuged for 5 min at 12 000rpm. Finally the supernatant was air dried on the bench for 15-45 min.
